# Editorial: Optimizing revascularization and conservative therapy in chronic coronary syndrome

**DOI:** 10.3389/fcvm.2025.1764352

**Published:** 2026-01-28

**Authors:** Josip Andelo Borovac, Dejan Milasinovic, Aleksandra Gasecka, Dino Miric

**Affiliations:** 1 Division of Ischemic Heart Diseases, Department of Cardiovascular Diseases, University Hospital of Split, Split, Croatia; 2 Department of Cardiology, University Clinical Center of Serbia, Belgrade, Serbia; 3 1st Chair and Department of Cardiology, Medical University of Warsaw, Warsaw, Poland

**Keywords:** ACS, acute coronary syndromes, CABG, CCS, chronic coronary syndrome, chronic total occlusions, coronary artery bypass grafting, CTO

## Introduction

Chronic coronary syndrome (CCS) is the most prevalent form of cardiovascular disease (CVD) and represents the leading cause of disability-adjusted life years and deaths worldwide, according to the latest update from the Global Burden of Disease (GBD) (1). In contrast to acute coronary syndromes (ACS), where early revascularization clearly improves survival and other clinical outcomes, the benefit of percutaneous coronary intervention (PCI) or coronary artery bypass grafting (CABG) beyond symptom relief and quality of life improvement in CCS is less certain. Relevant international guidelines emphasize optimal medical therapy (OMT) in all patients and carefully indicate revascularization for prognostically relevant anatomical substrates, impaired left ventricular systolic function, or refractory and limiting angina symptoms despite OMT (2, 3). The clinical reality, however, is a large grey zone where ischemia burden, anatomy, comorbidities, and patient preference must be balanced together to devise an individual and patient-tailored treatment (4, 5). The contemporary personalized approach to CCS should evaluate all aspects of interventions that integrate revascularization, medical therapy, and lifestyle interventions (6, 7).

The *Frontiers in Cardiovascular Medicine* Research Topic entitled “*Optimizing Revascularization and Conservative Therapy in Chronic Coronary Syndrome*” brings together an impressive collection of fifteen articles that collectively advance the field. This editorial synthesizes their main messages around four themes: (1) diagnosis and risk stratification, (2) use and timing of revascularization, (3) technical and procedural optimization, and (4) refinement of conservative therapy.

### Refining diagnosis and risk stratification

Several articles explored a fundamental question: which CCS patients truly warrant invasive evaluation and potential revascularization? Zhao et al. introduce ΔGCW, the change in global constructive work derived from strain echocardiography, as an early marker of ischemic risk. Combined with hemoglobin levels, ΔGCW improves discrimination of patients with functionally relevant ischemia, outperforming more conventional echocardiographic indices. This illustrates how advanced echocardiographic indices may refine selection for downstream testing in a non-invasive fashion. Another article by Tremamunno et al. reminds as all that simple clinical tools still matter, showing how exercise ECG may provide a value in CCS. In patients with suspected CCS, a clearly normal or low-risk treadmill test effectively ruled out left main coronary disease on subsequent angiography. In appropriate patients, a reassuring exercise test can thus support continued conservative management and avoid reflexive referral to the catheterization laboratory. Furthermore, Kong et al. examined the use of cardiopulmonary exercise testing (CPET) and demonstrated that lower heart rate at the anaerobic threshold and at the respiratory compensation point correlate with the presence of obstructive coronary artery disease (CAD) even in patients who do not reach maximal effort. These submaximal CPET parameters may therefore serve as surrogate markers of impaired chronotropic response when conventional exercise capacity is limited.

Taken together, these original studies illustrate how an integrated approach to functional assessment—from ECG stress testing through CPET to advanced echocardiographic strain imaging—can better discriminate those CCS patients who truly warrant invasive angiogram and potential revascularization from those who can safely remain on optimized medical therapy and undergo conservative pathway.

### Guiding and timing of coronary revascularization

Once we decide to proceed with coronary revascularization, several questions arise, such as how and when to do carry out the procedure.

A network meta-analysis by Liu et al. compared PCI guided by angiography alone, invasive physiology, and intravascular imaging in patients with acute coronary syndromes. Both fractional flow reserve (FFR)-guided and intravascular ultrasound (IVUS)-guided PCI were associated with fewer major adverse cardiovascular events than angiography-guided PCI, with IVUS emerging as the top-ranked strategy. Although focused on ACS, these data suggest that, when we decide to treat, doing so by using physiology and imaging yields better long-term results than relying on the angiography cines alone. Similarly, two manuscripts focused on the timing and completeness of revascularization. He et al. pooled randomized trials comparing immediate vs. staged multivessel PCI in ACS, thus showing that immediate complete revascularization reduced myocardial infarction and repeat revascularization without increasing mortality, supporting a one-sitting strategy in carefully selected, hemodynamically stable patients. Traditionally challenging patients are those with chronic total occlusions (CTO), and decision-making in this patient population is challenging (8). Maestre-Luque et al. provided findings with an observational series in CCS patients with chronic total occlusion (CTO), in whom angiographic complete revascularization, including successful CTO PCI, was associated with fewer mid-term adverse events such as residual ischemia. The symptomatic dimension of CTO PCI is examined by Will et al., who showed that successful CTO recanalization provided consistent outcome improvements by significantly reducing angina frequency and nitrate use, even in the absence of a demonstrable survival advantage. Finally, Bosnjak et al. remind us that revascularization is only part of the story. In patients with stable CAD who underwent revascularization, elevated levels of NT-proBNP and Galectin-3 after the procedure identified a subgroup at higher risk of future events. Persistent biomarker activation despite successful intervention on coronaries likely reflects a diffuse or cardiomyopathic substrate and points to the need for intensified heart-failure–directed therapies. Revascularization in HF remains particularly challenging and recent expert consensus suggests careful and multimodal evaluation of these patients (9). It also opens a research avenue: can biomarker-guided post-PCI strategies further improve outcomes in CCS?

### Technical nuances and procedural safety of coronary revascularization

A third cluster of manuscripts addresses practical challenges once the guide catheter is in the coronary ostium.

Wu et al. explore the impact of a myocardial bridge overlying an LAD CTO. Using IVUS-guided PCI, authors demonstrate that the presence of a bridge portends higher rates of restenosis, target lesion revascularization, and major adverse events at follow-up. An intramyocardial segment subjected to repetitive systolic compression appears to be an inherently hostile environment for stents. This argues for meticulous planning, careful stent sizing and expansion, and, where feasible, strategies that avoid extensive stenting within the bridged segment. Side-branch occlusion in LAD/diagonal bifurcation PCI is the focus of a validation study of the V-RESOLVE score. Wu et al. confirm that side-branch loss, although relatively infrequent, is strongly associated with worse clinical outcomes. High V-RESOLVE scores, driven by adverse bifurcation anatomy and limited side-branch protection, identified cases at high risk. Importantly, the underuse of intracoronary imaging was also linked to these adverse events. We now know that intravascular imaging during PCI improves safety and efficacy of the procedure, thus significantly reducing risks of death, MI, repeat revascularization, and stent thrombosis (10). Furthermore, a study by Xi et al. exemplifies the use of a risk score to trigger more protective strategies, including systematic wiring of the side branch, provisional or planned two-stent techniques, and liberal use of intravascular imaging. At the access-site level, Wang et al. provide data on immediate removal of brachial artery sheaths after PCI. By reversing approximately half of the procedural heparin dose with protamine, operators were able to remove sheaths at the end of the procedure without increasing major bleeding, while maintaining a low incidence of pseudoaneurysm formation. For centers that still use brachial access, this protocol can simplify post-procedural care and shorten immobilization, provided that local surveillance is maintained.

### Optimizing conservative therapy around revascularization

Revascularization decisions in CCS are inseparable from the background of OMT. One trial in this collection, by Wang et al., examines the combination of ticagrelor and extended-release metoprolol in elderly patients after PCI for ACS. Compared with standard care, the combination therapy improved left ventricular function, exercise capacity, and quality-of-life scores and was associated with more favorable profiles of inflammatory and myocardial injury biomarkers. These findings reinforce existing guideline recommendations on dual antiplatelet therapy and beta-blockade while emphasizing that elderly patients, who are often undertreated, can derive substantial functional benefit from combined cardioprotective therapies. More broadly, the special issue underscores that “conservative therapy” is anything but a mere passive concept. Across the articles, meticulous risk factor control, anti-ischemic medication, and HF-directed therapies remain the bedrock upon which any revascularization strategy rests. The question is rarely “stent or pills?” but rather “which patient, at which time, gains incremental benefit from an invasive strategy on top of already optimized medical care?”

### A look ahead: research gaps and future clinical implications

Diagnostic pathways for CCS will likely become more integrated and will involve multimodal imaging and biomarker stratification in the future. As we see in this Special Collection, exercise ECG, CPET-derived heart-rate indices, and advanced echocardiographic measures such as ΔGCW each contribute complementary information. The key research need is to define practical algorithms that combine these tools in a cost-effective, patient-centered way and that can be implemented beyond tertiary centers. When revascularization is elected as a treatment option, the evidence increasingly favors doing it completely and doing it well: complete multivessel PCI in stable ACS patients, pursuit of complete revascularization in suitable CTOs, and liberal use of intravascular imaging and physiology help us make the right decisions and optimize stent deployment. Randomized data specific to CCS, particularly in patients with extensive comorbidities or complex anatomy, remain limited and should be a priority for future clinical trials.

In conclusion, the 15 articles in this Research Topic move us beyond the simplistic dichotomy of “revascularization vs. conservative therapy”. Instead, they support a more nuanced vision: CCS as a disease continuum in which high-quality diagnostic modalities, personalized decisions about timing and completeness of revascularization, technical excellence in the cath lab, and rigorous optimization of medical therapy are interlocking components. For clinicians, this means fewer automatic reflexes and more thoughtful, evidence-based conversations with patients.
